# Alternative splicing derived invertebrate variable lymphocyte receptor displays diversity and specificity in immune system of crab *Eriocheir sinensis*

**DOI:** 10.3389/fimmu.2022.1105318

**Published:** 2023-03-14

**Authors:** Yuanfeng Xu, Yanan Yang, Jinbin Zheng, Zhaoxia Cui

**Affiliations:** ^1^ School of Marine Sciences, Ningbo University, Ningbo, China; ^2^ Laboratory for Marine Biology and Biotechnology, Qingdao National Laboratory for Marine Science and Technology, Qingdao, China

**Keywords:** variable lymphocyte receptor, alternative splicing, specific, diverse, immune system, *Eriocheir sinensis*

## Abstract

Variable lymphocyte receptors (VLRs) play vital roles in adaptive immune system of agnathan vertebrate. In the present study, we first discover a novel VLR gene, VLR2, from an invertebrate, the Chinese mitten crab, *Eriocheir sinensis*. VLR2 has ten different isoforms formed *via* alternative splicing, which is different from that in agnathan vertebrate with the assembly of LRR modules. The longest isoform, VLR2-L, responds to Gram-positive bacteria *Staphylococcus aureus* challenge specifically, while shows no response to Gram-negative bacteria *Vibrio parahaemolyticus* challenge, confirmed by recombinant expression and bacterial binding experiments. Interestingly, VLR2s with short LRRs regions (VLR2-S8 and VLR2-S9) tend to bind to Gram-negative bacteria rather than Gram-positive bacteria. Antibacterial activity assay proves six isoforms of VLR2 have pluralistic antibacterial effects on bacteria which were never reported in invertebrate. These results suggest that the diversity and specificity of VLR2 resulted from alternative splicing and the length of the LRRs region. This pathogen-binding receptor diversity will lay the foundation for the study of immune priming. Furthermore, studying the immune function of VLR2 will provide a new insight into the disease control strategy of crustacean culture.

## Introduction

Variable lymphocyte receptor (VLR) is a core immune factor in adaptive immune system of agnathan vertebrate. It is first discovered in *Lampetra japonicum* (1, 2). VLR is mainly composed of leucine-rich repeat (LRR) modules, and has two states: germline VLR (Germ variable lymphocyte receptor, gVLR) and mature VLR (1). The latter has diversity and can specifically bind to different antigens (1). The diversity of mature VLR genes is formed by the assembly of LRR modules, resulting in at least 10^14^ unique antigen-specific receptors, and the degree of mature VLR diversity is comparable to that of mammalian antibody diversity (3). A large number of different mature VLRs act as antigen receptors that specifically bind to various pathogens and enhance adaptive immune responses by activating the complement activation cascade (1, 4). In *Eptatretus burgeri*, the specific VLR against Nervous Necrosis Virus (NNV) reduces the infectivity effectively (5), another specific VLR against Viral Hemorrhagic Septicemia Virus (VHSV) is modified to work as a diagnostic tool for VHSV (6). Subsequently, VLR-like genes are also found in *Branchiostoma floridae*, while no LRR-box rearrangement assembly mechanism similar to that in agnathan vertebrate is detected (7).

In recent years, VLR genes or VLR-like genes have been found in invertebrate, such as *Apostichopus japonicus*, *Litopenaeus vannamei*, *Eriocheir sinensis*, etc. (8–10). In *E. sinensis*, the VLR gene exhibits strong bacteria-binding activity rather than antibacterial activity, and functions in crab immune priming through its immune memory-like characteristic (10). The above studies mainly focus on gene structure analysis and immune functions of VLR and VLR-like genes under pathogen challenge. However, it is still uncertain whether the VLR genes in invertebrate have diversity. If there is diversity of invertebrate VLRs, the formation mechanism and biological significances of this diversity also remain unknown.

As the model animal with easy culture and transportation of crustacean, the Chinese mitten crab, *E. sinensis* is an important freshwater cultured economic species. With the continuous expansion of breeding scale, large-scale diseases caused by bacteria, viruses and other pathogens also break out frequently causing huge losses to the industry of *E. sinensis* (11–14). Similar to other invertebrate sepcies, the *E. sinensis* is considered to own only the innate immune system, which can resist the invasion of foreign pathogens through humoral immunity and cellular immunity (15, 16). *E. sinensis* lacks an adaptive immune system and cannot produce diverse and specific antibodies against the invasion of foreign pathogens through Ig/TCR gene recombination (17). The recent studies show 30,600 Down syndrome cell adhesion molecule (Dscam) isoforms, which are generated *via* alternative splicing, can bind specifically with the original bacteria to facilitate efficient clearance in *E. sinensis* (18). Therefore, it is necessary to discover if more immune genes have the genetic diversity.

In this study, a novel VLR gene, VLR2, is discovered from *E. sinensis*. VLR2 has ten different isoforms, which are formed *via* alternative splicing. Recombinant expression, bacterial challenge and bacterial binding experiments are adopted to prove different isoforms own specific bacterial binding ability. This study should propose a new mechanism for the formation of VLR diversity and discover the biological significance of the diversity in the immune system of invertebrate. The diversity and specific bacterial binding ability of VLR2 provide insights for further studying the role of VLR in immune priming.

## Materials and methods

### Animal and sample preparation

Healthy male *E. sinensis* (100 ± 5 g) purchased from Sanshan market in Ningbo, China. All crabs were acclimated in tanks with filtered freshwater at room temperature (20 - 26°C) for one week before processing. Crab hemolymph was harvested from the last walking leg with an equal volume of ice-cold anticoagulant buffer (27 mM sodium citrate, 336 mM NaCl, 115 mM glucose, 9 mM EDTA, pH = 7.0), then immediately centrifuged at 800 g, 4°C for 5 min to isolate hemocytes. Crab tissues were collected after being euthanized in accordance with the Institutional Animal Care and Use Committee (IACUC) guidelines.

### RNA isolation, cDNA synthesis and gene cloning of VLR2s

The total RNA was extracted using TRIzol reagent (Invitrogen) and the cDNA was synthesized using a PrimeScript™ RT Reagent Kit (Takara) according to the manufacture’s protocols. The raw data of the full-length transcriptome of the hepatopancreas of *E. sinensis* was downloaded from the NCBI database (accession number: SRR12632765). After the quality control and filter of raw reads, full-length reads were aligned to the *E. sinensis* genome (accession number: LQIF00000000) using the Hierarchical Indexing for Spliced Alignment of Transcripts (HISATS2) (19). Then, the aligned files was visualized using the Integrative Genomics Viewer (IGV) (20), and isomforms of VLR2 were observed in IGV. Based on these conserved regions in 5′-UTR and 3′-UTR of isomforms, a set of primers (VLR2-F and VLR2-R, **Table 1**) were designed to amplify the open reading frames (ORFs) of VLR2’s alternative splicing transcripts which span 5′-UTR to 3′-UTR. The PCR was performed in a 20 μL reaction volume consisting of 13.1 μL of sterile distilled H_2_O, 2.0 μL of 10 × PCR buffer,1.6 μL of dNTP (10 mM), 0.5 μL of each primer (10 mM), 0.3 μL (1 U) of Easy Taq polymerase (TransGen) and 2 μL of DNA template. The PCR programs were set as follows: 94°C for 2 min, followed by 32 cycles of 94°C for 30 s, 50°C for 30 s, 72°C for 1.5 min, and a final extension at 72°C for 5 min. The PCR products were analyzed by 1.2% agarose gel electrophoresis and purified by PCR purification kit (Axygen) as described in manufacture. The purified PCR products were cloned into the pMD19-T simple vector (TaKaRa). After being transformed into the competent cells of *Escherichia coli* DH5α, the positive recombinants were identified through the anti-Amp selection and PCR screening with M13-47 and RV-M primers (**Table 1**). Clones with different inserts were sequenced and the ORFs of VLR2′s alternative splicing transcripts were confirmed by aligning with VLR2 sequence among genome.

**Table 1:** Primer sequences used in the present study.

Primer name	Primer sequence (5′-3′)	Target
**VLR2-F**	TGGTGGTGATGATGGGTC	cDNA fragment
**VLR2-R**	ACATCCCTGCTCCCAC	cDNA fragment
**M13-47**	CGCCAGGGTTTTCCCAGTCACGAC	cDNA fragment
**RV-M**	GAGCGGATAACAATTTCACAGG	cDNA fragment
**VLR2L-QF**	CACCCTGGAGCAGATGA	qRT-PCR
**VLR2L-QR**	GACTCCTTGCCATTCTTTAC	qRT-PCR
**VLR2G-QF**	TGAAGATGTGGTGCTCGCAT	qRT-PCR
**VLR2G-QR**	TGAAAACACTCCCCGGAAGG	qRT-PCR
**ACTIN-QF**	GCATCCACGAGACCACTTACA	qRT-PCR
**ACTIN-QR**	CTCCTGCTTGCTGATCCACATC	qRT-PCR
**T7F**	TAATACGACTCACTATAGGG	Protein expression
**T7R**	TGCTAGTTATTGCTCAGCGG	Protein expression
**VLR2_RE-F**	CGCGGATCCTGGTGGTGATGATGGGTC	Protein expression
**VLR2_RE-R**	CCCAAGCTTACATCCCTGCTCCCAC	Protein expression

### Sequence and phylogenetic analysis

The deduced amino acid sequence was analyzed with Tbtools and visualized by DNASTAR. SMART (http://smart.embl-heidelberg.de/) and NCBI BLAST program (https://blast.ncbi.nlm.nih.gov/Blast.cgi/) were used to predict the structures and functional domains of VLR2 isoform proteins. The Protein Analysis Tools (http://www.bio-soft.net/sms/) was performed to predict the molecular weight (Mw) and theoretical isoelectric point (pI) of the protein. SignalP 5.0 (https://services.healthtech.dtu.dk/service.php?SignalP-5.0) was used to predict signal peptides and TMHMM Server v.2.0 (https://services.healthtech.dtu.dk/service.php?TMHMM-2.0) was used for transmembrane regions prediction. The three-dimensional structure models were built by SWISSMODEL (https://swissmodel.expasy.org/) and visualized by Swiss-PdbViewer.

### Semi-quantitative PCR

Semi-quantitative PCR (Semi-qPCR) was applied to study the tissue expression pattern of VLR2 isoforms using two pairs of gene-specific primers (**Table 1**). VLR2L-QF and VLR2L-QR primers were specific to VLR2-L, VLR2G-QF and VLR2G-QR primers were suitable to all VLR2 isoforms including VLR2-L. The procedure of Semi-qPCR is the same as the PCR process as described above.

### Challenge assays

The challenge assays were according to the previous method (21) with some modification. *Vibrio parahaemolyticus* and *Staphylococcus aureus* during logarithmic growth phase were centrifuged at 800 × g, 4°C for 10 min, and washed three times with sterile 0.1 M phosphate buffer saline (PBS, pH = 7.4). Finally, *V. parahaemolyticus* and *S. aureus* were diluted to 10^7^ CFU/mL with PBS.

During the challenge experiment, 30 crabs were injected with 50 μL *V. parahaemolyticus* (1 × 10^7^ CFU/mL, named as VP group), 30 crabs were injected with 50 μL *S. aureus* (1 × 10^7^ CFU/mL, named as SA group), and the control group was injected with the equal volume of PBS (named as PBS group). At 3, 6, 12 and 24 h post-injection, six individuals from each group were randomly sampled to collect tissues. Then, tissues were used for RNA isolation and cDNA synthesis as described above.

### Quantitative real-time PCR analysis of VLR2s

Quantitative real-time PCR (qRT-PCR) was applied to study the expression alterations of VLR2 isoforms in *E. sinensis* after challenge assays. Reactions were carried out on an ABI PRISM 7500 Sequence Detection System (Applied Biosystems) using SYBR green II (TaKaRa) as the fluorescent dye. The β-actin was chosen as a reference gene for internal standardization. The PCR reaction was carried out in a total volume of 20 μL, containing 10 μL of TB Green Premix DimerEraser (TaKaRa), 0.4 μL of 50 ROX Reference Dye II (TaKaRa), 2 μL of the diluted cDNA, 0.6 μL of each primer (10 μM), and 6.4 μL of sterile distilled water. The PCR program was 95°C for 5 min, followed by 40 cycles of 95°C for 5 s and 55°C for 31 s. Dissociation curve analysis of the amplification products was performed at the end of each PCR reaction to confirm that only one PCR product was amplified. All samples were repeated in triplicates in the qRT-PCR analysis. Fold change for the gene expression relative to controls was determined by the 2^-△△Ct^ method (22).

### The construction of recombinant plasmids

The ORFs of the VLR2 variants (VLR2-L, VLR2-S2, VLR2-S3, VLR2-S5, VLR2-S8 and VLR2-S9) were amplified by PCR using a pair of primers containing BamH I and EcoR I sites (VLR2_RE-F and VLR2_RE-R, **Table 1**). The PCR products were analyzed by 1.2% agarose gel electrophoresis. Then the PCR products were purified by PCR purification kit (Axygen) and cloned into the pMD19-T simple vector (TaKaRa) as described in manufacture.

The recombinant plasmids (pMD19-T-VLR2s) and pET-32a (+) vector were digested with the restriction enzymes BamH I and EcoR I (Thermo), then the PCR products were inserted into pET-32a (+) vector. Afterward, the recombinant plasmids (pET-32a-VLR2s) were transformed into the Rosetta cells (ANG YU BIO) for vitro expression. The pET-32a (+) vector without insert fragment was selected as a negative control.

### Expression and purification of recombinant VLR2s

Overnight culture of a single colony of Rosetta cells was diluted (1:100) with Luria-Bertani medium and incubated at 37°C with vigorous shaking to OD600 of 0.4–0.6. Isopropyl β-D-1-Thiogalactopyranoside (IPTG) was added at a final concentration of 0.2 mM to induce the protein expression for a period of 4 h at 25°C. The protein extract was analyzed by 15% (w/v) SDS-PAGE.

The cells were broken by supersonic wave at 0°C and centrifuged at 12,000 × g, 4°C for 30 min. The soluble fractions and insoluble fractions of broken cells were analyzed by 15% (w/v) SDS-PAGE. The supernatants containing recombinant proteins were collected for subsequent purification experiments and the precipitations containing recombinant proteins were denatured and renatured as previously reported (23) to obtain soluble proteins. The supernatant and soluble proteins were loaded on to a Ni-nitrilotriaceticacid-agarose affinity column (Novagen). The recombinant protein was eluted with four concentration of elution buffers (20 mM Tris pH 8.0, 500 mM NaCl, 50/100/200/500 mM imidazole) and dialyzed in PBS at 4°C overnight. The extracted protein was analyzed by 15% (w/v) SDS-PAGE and Western blot.

### Assay of binding activity to bacteria

An equal volume of bacteria solution (0.5 mL, 3 × 10^8^ CFU/mL) with PBS (control group) and purified proteins (final concentration, 1 mg/mL proteins) were mixed and incubated with gentle rocking at 4 °C for 30 min respectively. After centrifugation at 800 × g and 4 °C for 5 min, the supernatant was removed, and the cells were washed twice with PBS. The supernatant, washed and eluted fractions were analyzed by 15% (w/v) SDS-PAGE.

### Antibacterial activity assay of the recombinant proteins

Bacteria suspended in PBS at a concentration of 2.6 × 10^7^ CFU/mL was used for the antibacterial activity test. Fifty microliters of recombinant proteins (250 μg/mL) were mixed with the same volume of bacteria solution and incubated at 28°C for 2h respectively. Then, the mixtures were diluted (10^4^ fold) and spread on Luria-Bertani or 2216E agar plates, and the CFU were calculated after culturing for 16 h at 28°C or 37°C. PBS and pET-32a were used as a blank control and negative control, respectively, and each group was repeated three times.

### Statistical analysis

Statistical analyses were processed using SPSS 26.0 software. Data obtained from this study were presented as the mean ± standard deviation (S.D.). Data for challenge assays and antibacterial activity assay were first subjected to Levene’s test for testing the homogeneity of variances and then subjected to one-way analysis of variance (ANOVA) with Scheffé’s method *post hoc* analysis. Differences were considered statistically significant at P < 0.05.

## Results

### Sequence analysis of VLR2s

**Figure 1:**
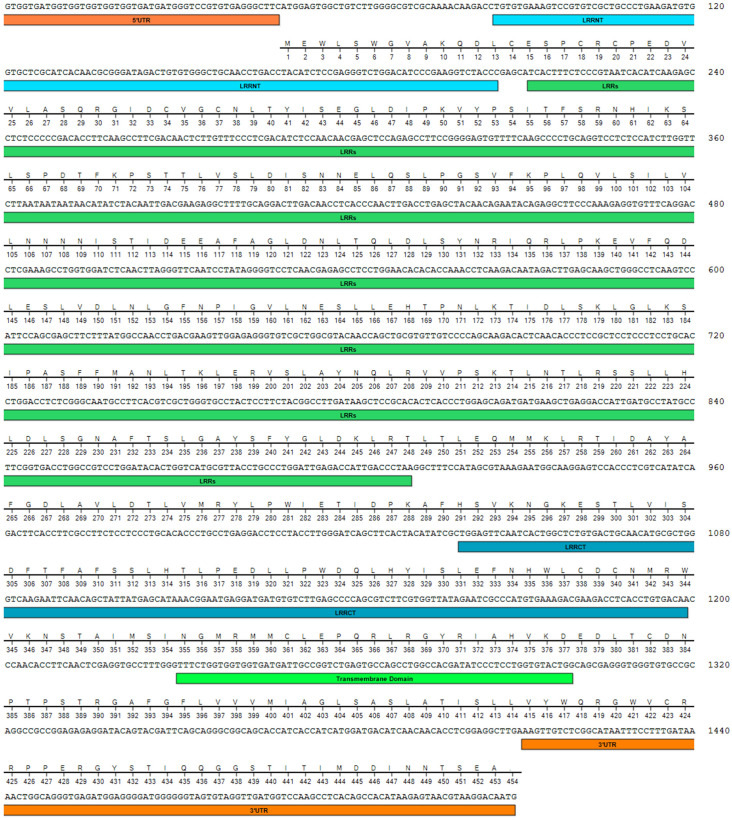
Nucleotide and deduced amino acid sequences of VLR2-L. The 5′-UTR and 3′-UTR were marked with orange, and the LRRNT, LRRs, LRRCT and transmembrane domain were marked with lightblue, green, navyblue and lightgreen respectively.

**Figure 2:**
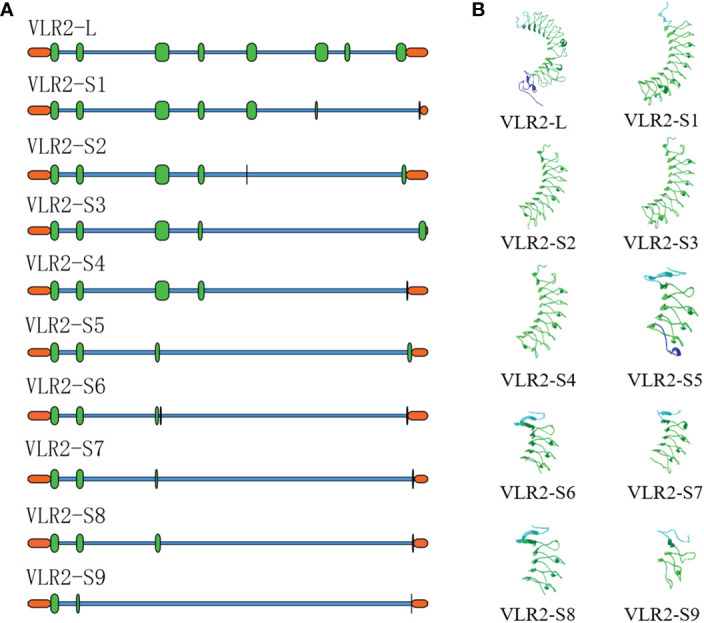
Sequence and structure analysis of VLR2 isoforms. **(A)**. Multiple alignments of the isoforms to *Eriocheir sinensis* genome. The orange modules indicated UTRs, the green modules indicated exons and the blue lines indicated introns. **(B)**. Three-dimensional structure prediction of VLR2 isoforms.

Ten ORF sequences of VLR2 isoforms were amplified and verified, which were classified into two types: long-type and short-type. The longest isoform, which was 1529 bp in ORF length, belongs to long-type and was termed as VLR2-L. While, other isoforms ranged from 309 to 801 bp in ORF length, were grouped into short-type and were termed as VLR2-S1 to VLR2-S9 according to ORF length. The VLR2-L encoded a 453-amino-acid-long protein with a predicted molecular weight of 50.81 kDa and a theoretical isoelectric point of 5.45 (**Figure 1**). VLR2-L contained LRR_NT, LRRs, LRR_CT and transmembrane domains, which were conserved among invertebrate VLRs. However, no transmembrane domain was found in VLR2-S1 to VLR2-S9, which only contained parts of LRR_NT, LRRs and LRR_CT domains. As shown in **Figure 2A**, all VLR2 isoforms were located in the same region of *E. sinensis* genome and were formed *via* alternative splicing. In addition, the crescent-shaped VLR solenoid structure, considered as a notable feature of VLR and resulting in highly specific binder, was detected in all VLR2 isoforms (**Figure 2B**). These ten ORF sequences were available at NCBI GenBank under accession numbers OP850588 - OP850597.

### Expression pattern analysis

**Figure 3:**
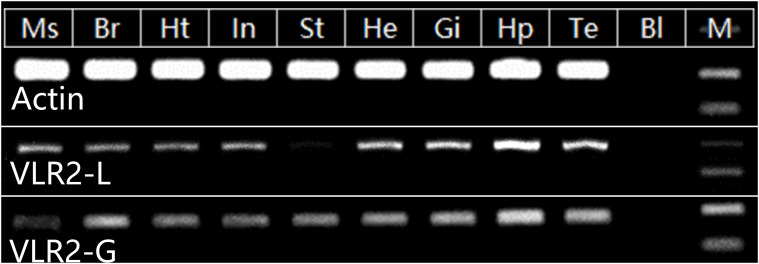
Tissue distribution of VLR2. Ms, muscle; Br, brain; Ht, heart; In, intestine; St, stomach; He, hemocytes; Gi, gill; Hp, hepatopancreas; Te, testis; Bl, blank; M, DL2000 Marker.

A broad spatial expression profile for VLR2-L and VLR2-G (all VLR2 isoforms including VLR2-L) was generated from tissues using Semi-qPCR (**Figure 3**). The hepatopancreas was considered as primary tissue responsible for both VLR2-L and VLR2-G expression. Interestingly, VLR2-L and VLR2-G performed consistent expression profile among most tissues except the stomach, where VLR2-G showed significantly higher expression than VLR2-L. This phenomenon indicated VLR2 was expressed as the long-type (VLR2-L) predominantly among most tissues except the stomach, while in the stomach, the predominantly-expressed VLR2 type was short-type (VLR2-S1 to VLR2-S9). The raw images of expression pattern analysis were available at **Supplementary File 1** (**Figures S1**, **S2**).

### Expression patterns in response to bacterial challenge

**Figure 4:**
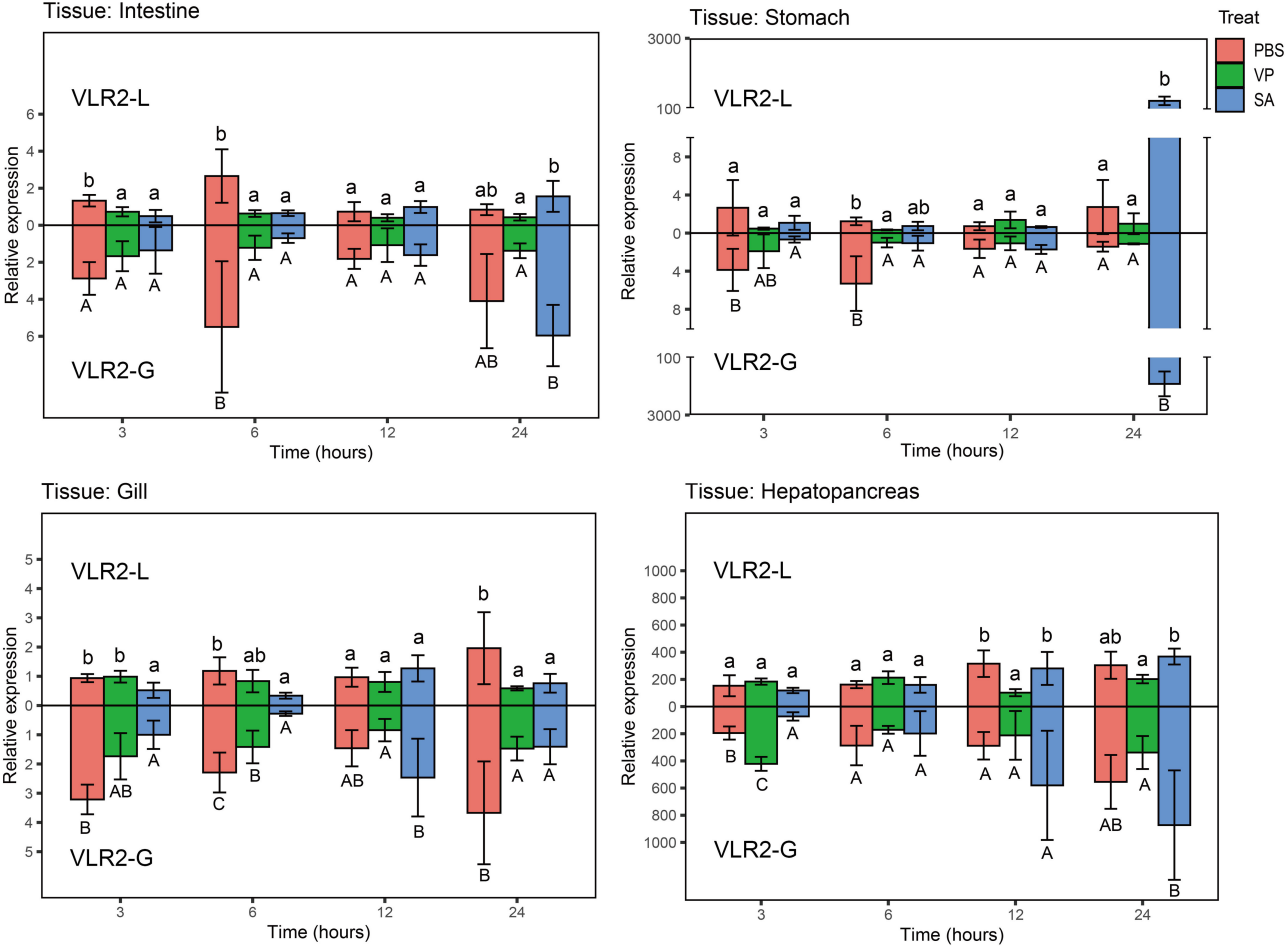
Expression profiles of VLR2 in immune-related tissues of *E. sinensis* challenged with *Vibrio parahaemolyticus* and *Staphylococcus aureus*. Each bar represented the mean ± SD (*n* = 4). Significant differences of the same group at p < 0.05 (*n* = 4, ANOVA) in different treatments were indicated by different letters while the same letters indicated no significant difference.

To obtain preliminary knowledge on the immune function of VLR2s in response to pathogen challenge, the expression profiles of VLR2s in intestines, stomachs, gills and hepatopancreas of *E. sinensis* post-injection with *V. parahaemolyticus* and *S. aureus* were investigated (**Figure 4**; **Supplementary File 2**). The results showed that VLR2s responded differently to *V. parahaemolyticus* and *S. aureus*. During *V. parahaemolyticus* challenge, except in the early stage of injection (3 and 6 h post-injection), the transcription of VLR2s in intestines and stomachs decreased slightly, and no significant transcriptional change of VLR2s occurred in other cases, which indicated that VLR2s were insensitive to *V. parahaemolyticus*. During *S. aureus* challenge, the transcription of VLR2s tended to decrease at 3 and 6 h post-injection, then started to increase and exceeded the basal level at 12 and 24 h post-injection. Surprisingly, the transcription of VLR2s in stomachs dramatically increased at 24 h post-injection, and the increase of transcription in stomachs was much more than that in other tissues. Above results implied that VLR2s participated in the defense against *S. aureus* and the stomach might be the vital tissue where VLR2s played roles. In addition, the expression profiles of VLR2-L post-injection were consistent with that of VLR2-G basically, and transcript levels of VLR2-L were slightly lower than VLR2-G generally, suggesting VLR2-L was predominantly-expressed among VLR2s.

### Bacteria-binding activity

**Figure 5:**
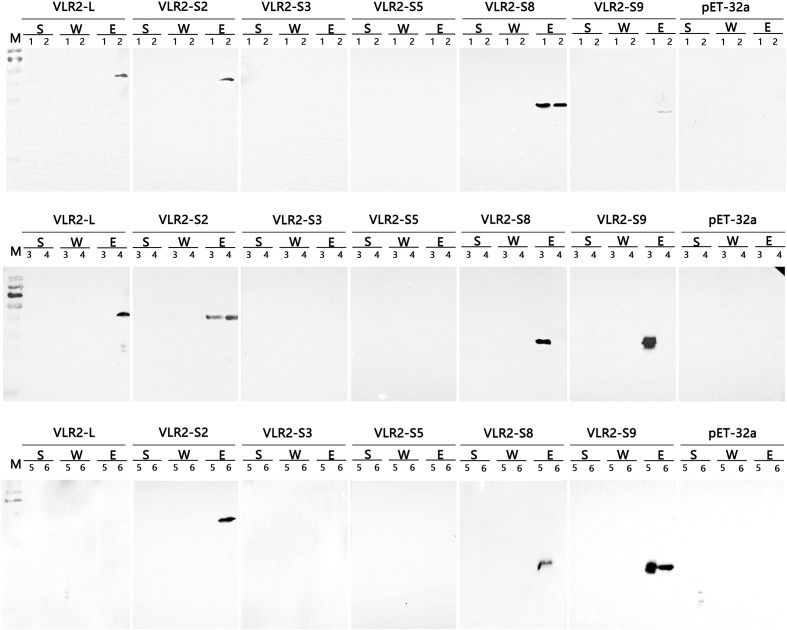
Binding activity of the recombinant VLR2 isoforms to Gram-negative bacteria *V. parahaemolyticus* (1), *Vibrio alginolyticus* (3) and *Vibrio harveyi* (5), and Gram-positive bacteria *S. aureus* (2), *Corynebacterium glutamicum* (4) and *Micrococcus lysodeikticus* (6). S, the supernatants separated by centrifugation after incubation; W, the waste liquid during washing cells; E, the precipitations eluted with PBS after centrifugation and washing.

To investigate the specific bacteria-binding ability of VLR2 isoforms, six recombinant VLR2s (rVLR2-L, rVLR2-S2, rVLR2-S3, rVLR2-S5, rVLR2-S8 and rVLR2-S9) were adopted to bind three Gram-positive bacteria (*S. aureus, Corynebacterium glutamicum* and *Micrococcus lysodeikticus*) and three Gram-negative bacteria (*V. parahaemolyticus, Vibrio alginolyticus* and *Vibrio harveyi*) (**Figure 5**). The rVLR2-L bound with two Gram-positive bacteria (*S. aureus* and *C. glutamicum*) and no Gram-negative bacterium, and rVLR2-S2 bound with three Gram-positive bacteria (*S. aureus*, *C. glutamicum* and *M. lysodeikticus*) and only one Gram-negative bacterium (*V. alginolyticus*). The rVLR2-S8 bound with three Gram-negative bacteria (*V. parahaemolyticus, V. alginolyticus* and *V. harveyi*) and only one Gram-positive bacterium (*S. aureus*), and rVLR2-S9 bound with three Gram-negative bacteria (*V. alginolyticus* and *V. harveyi*) and only one Gram-positive bacterium (*M. lysodeikticus*). No bacterium was detected to bind with two medium-length rVLR2s, rVLR2-S3 and rVLR2-S5. These results suggested that, the longest rVLR2s (rVLR2-L and rVLR2-S2) and the shortest rVLR2s (rVLR2-S8 and rVLR2-S9) were biased toward binding Gram-negative bacteria and Gram-positive bacteria respectively. We speculated that VLR2 isoforms exhibited the specific bacteria-binding activity might be related with amino acid length. The raw images of bacteria-binding activity were available at **Supplementary File 1** (**Figures S3**–**S13**).

### Anti-bacteria activity

**Figure 6:**
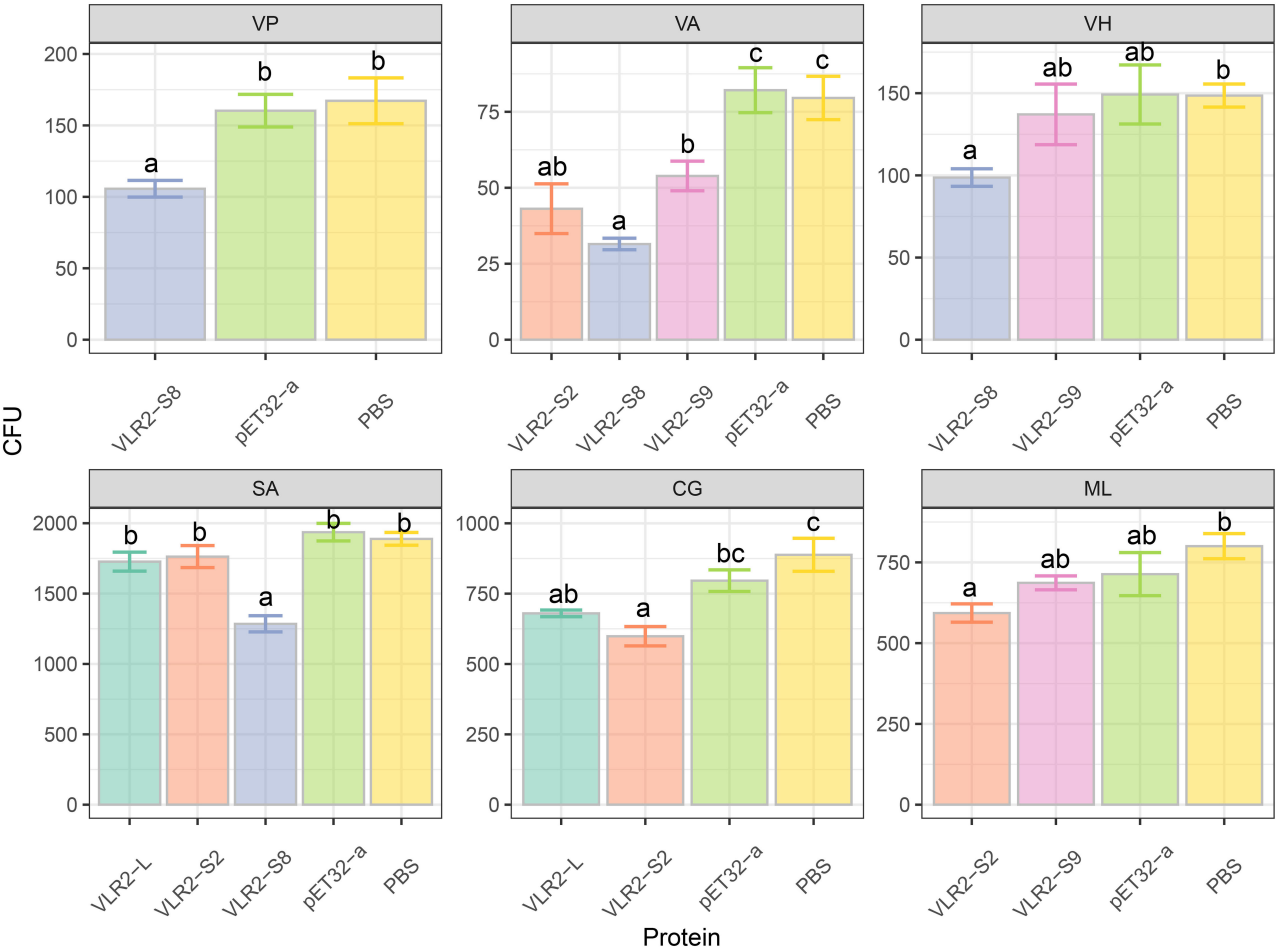
Anti-bacteria activity of the recombinant VLR2 isoforms to bound bacteria. Each bar represented the mean ± SD (*n* = 3). Significant differences of the same group at p < 0.05 (*n* = 3, ANOVA) in different treatments were indicated by different letters while the same letters indicated no significant difference.

The rVLR2s with specific bacteria-binding activity (rVLR2-L, rVLR2-S2, rVLR2-S8 and rVLR2-S9) were employed to test anti-bacteria activity against bound bacteria (**Figure 6**; **Table 2**; **Supplementary File 3**). Significant anti-bacteria activity of rVLR-S8 against all four bound bacteria (*V. parahaemolyticus, V. alginolyticus*, *V. harveyi* and *S. aureus*) was observed. The rVLR2-S2 and rVLR2-S9 exhibited significant anti-bacteria activity against parts of bound bacteria, the rVLR2-S2 was against *V. alginolyticus*, *C. glutamicum* and *M. lysodeikticus*, and rVLR2-S9 was against *V. alginolyticus* and *V. harveyi*. However, no significant anti-bacteria activity of rVLR2-L against bound bacteria was detected.

**Table 2:** Summary of anti-bacteria activity.

Protein Bacteria	VLR2-L	VLR2-S2	VLR2-S8	VLR2-S9
***V. parahaemolyticus***	NA	NA	**+**	NA
***V. alginolyticus***	NA	**+**	**+**	**+**
***V. harveyi***	NA	NA	**+**	**+**
***S. aureus***	**−**	**−**	**+**	NA
***C. glutamicum***	**−**	**+**	NA	NA
***M. lysodeikticus***	NA	**+**	NA	**−**

+, a significant anti-bacteria activity was detected in this combination; **−**, no significant anti-bacteria activity was detected in this combination; NA, anti-bacteria activity was not tested in this combination. Differences were considered statistically significant at p < 0.05 (n = 3, ANOVA).

## Discussion

VLR is a key immune factor in adaptive immune system of agnathan vertebrate and is first discovered in *L. japonicum* (1, 2). Recently, VLR genes or VLR-like genes are also identified in invertebrate and found probably to play a role in the invertebrate immune system (8–10). In this study, a novel VLR gene named VLR2 is identified in *E. sinensis*. VLR2 has multiple transcripts and performs a certain degree of diversity, which is similar to VLRs of agnathan vertebrate, rather than the reported invertebrate VLRs such as EsVLRA in *E. sinensis* (8), Aj-VLRA in *A. japonicas* (9) and LvLRRm in *L. vannamei* (10).

In agnathan vertebrate, there is a special formation mechanism of VLR diversity, when stimulated, the LRR modules on both sides of gVLR in lymphocytes will randomly insert into gVLR, and gradually form mature VLR with diversity (24). Although no similar assembly mechanism has been found in invertebrate, the alternative splicing can provide the genetic diversity (25), such as the Dscam in *Drosophila melanogaster* and *E. sinensis* (25, 26), the Toll-like receptor (TLR) genes TLR1, TLR2 and TLR3 in *L. vannamei* (27). In this study, the amplified ORFs of VLR2 are aligned with the *E. sinensis* genome and we find that ten isoforms are formed *via* alternative splicing. This is the first report of finding the diversity of VLRs in invertebrate, also suggests the mechanism of the diversity may be related to the alternative splicing.

LRR_NT, LRRs, LRR_CT, and transmembrane domains are the main features of VLRs, which present in both agnathan vertebrate and invertebrate (1, 8). Ten VLR2 isoforms in this study also have above structural characteristics, except that VLR2-S1 to VLR2-S9 do not contain transmembrane domains. The lengths of VLR2 isoforms (from 103 to 453 aa) are shorter than that of EsVLRA (799 aa) in *E. sinensis* (8) and Aj-VLRA (664 aa) in *A. japonicus* (9), which are closer to *Petromyzon marinus* VLRs (≤ 417 aa) (28). The three-dimensional structural model prediction shows that all ten VLR2 isoforms own crescent-shaped solenoid structures in LRRs regions (29) and these structures might perform microbial recognition functions (30), which has also been reported in EsVLRA (8).

VLR belongs to antigen receptors, which recognizes pathogens (8) and locates on the surface of cells in immune-related tissues (31). The hepatopancreas is considered as a critical immune tissue of crustaceans and the primary site for the production of immune recognition molecules (32). In this study, the hepatopancreas is the primary tissue responsible for VLR2 expression, which is consistent with the study of EsVLRA (8). Compared with other isoforms, almost no expression of VLR2-L is detected in the stomach. It suggests the immune function of VLR2-L may be different from short-type VLR2s (VLR2-S1 to S9) in *E. sinensis*.

Strangely, the experimental results of bacterial challenge show that, the transcription of VLR2-L in stomach increase sharply post *S. aureus* challenge, while remain steady post *V. parahaemolyticus* challenge. The dramatic expression change in stomach may be related to the function of the stomach as the first line against orally pathogenic infection (33). Furthermore, VLR2-L is detected to bind with *S. aureus*, rather than *V. parahaemolyticus*. Above findings imply that VLR2-L could defend against *S. aureus* specifically, which is comparable to VLRs with specific immune functions in agnathan vertebrate, including the *Bacillus anthracis* specific VLR in *L. japonicum* (3) and the NNV specific VLR in *E. burgeri* (5). In addition, we find that the isoforms with long LRRs regions (VLR2-L and VLR2-S2) tend to bind to Gram-positive bacteria, while the isoforms with short LRRs regions (VLR2-S8 and VLR2-S9) preferentially bind to Gram-positive bacteria. The specific binding mode of VLR2 isoforms is similar to that reported in Dscam, which has specific binding and scavenging ability for different pathogens (25). The relationship between binding mode and LRRs region length of VLR2s is similar to that reported in *E. burgeri* (5).

In agnathan vertebrate, VLRs bind to various pathogens specifically and exert the antibacterial effect by activating the complement activation cascade and regulating interleukins (1, 4). But, no VLR has been reported to perform anti-bacteria activity in invertebrate. In this study, we find that the VLR2 isoforms exhibit pluralistic anti-bacteria activity. The VLR2-S8 exert significant antibacterial effects on all bound bacteria, while VLR2-S2 and VLR2-S9 only perform significant antibacterial effects on parts of bound bacteria. Above results indicate that the anti-bacteria activities of VLR2 isoforms are pluralistic, which share similarities with VLRs of agnathan vertebrate to a certain extent. These VLR2s could be added to the feed to defend against bacteria of crabs (34).

## Conclusions

In conclusion, we identify a novel VLR gene named VLR2 in *E. sinensis* and this gene can produce ten different isoforms *via* alternative splicing. The VLR2 isoforms play complex immune functions, which show a certain degree of specificity in the immune system of *E. sinensis*, and this specificity could be related to the LRRs region lengths of isoforms. This study shows that VLR2 isoforms exert vital immune functions in *E. sinensis* immune system, and the pathogen-binding receptor diversity based on VLR2 lay the foundation for the study of immune priming. In addition, studying the immune function of VLR2 also provides a new insight into the disease control strategy of crustacean culture.

## Data availability statement

The data presented in the study are deposited in the NCBI repository, accession number OP850588 - OP850597 (https://www.ncbi.nlm.nih.gov/popset/?term=OP850588%20-%20OP850597).

## Author contributions

YX: Conceptualization, Methodology, Validation, Investigation, Writing–original draft. YY: Methodology, Validation, Investigation, Writing–review and editing. JZ: Methodology, Investigation. ZC: Resources, Funding acquisition, Writing–review and editing, Supervision. All authors contributed to the article and approved the submitted version.
