# Supplementary material for: Alternative splicing derived invertebrate variable lymphocyte receptor displays diversity and specificity in immune system of crab Eriocheir sinensis
Source: Front Immunol. 2023 Mar 14;13:1105318. doi: 10.3389/fimmu.2022.1105318 (PMC10045472; doi:10.3389/fimmu.2022.1105318)

Figure S1. The raw image of tissue distribution of Actin and VLR2-G.


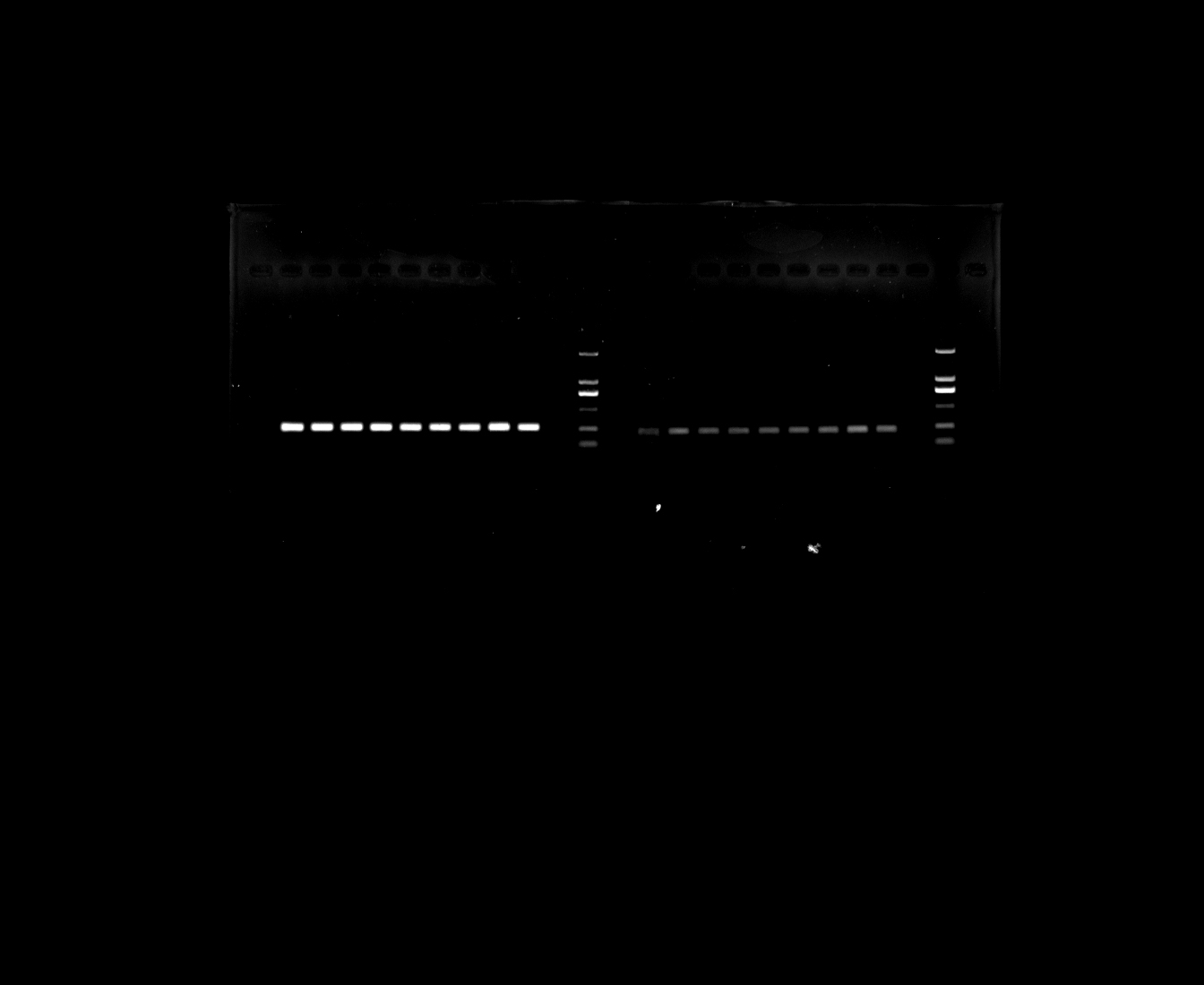


Figure S2. The raw image of tissue distribution of VLR2-L.


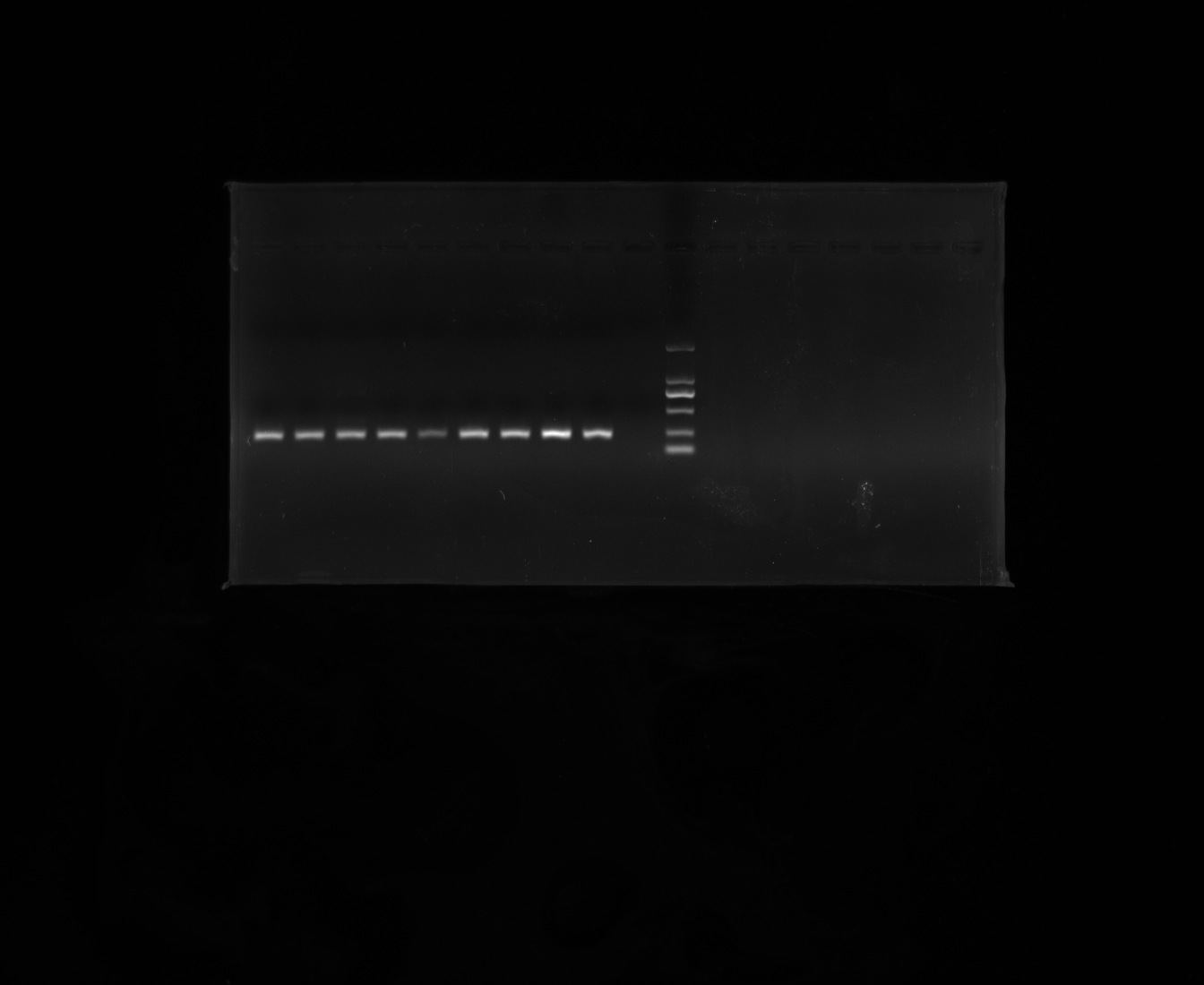


Figure S3. The raw image of binding activity of the recombinant VLR-L and VLR2-S3 to *Vibrio parahaemolyticus* and *Staphylococcus aureus*.


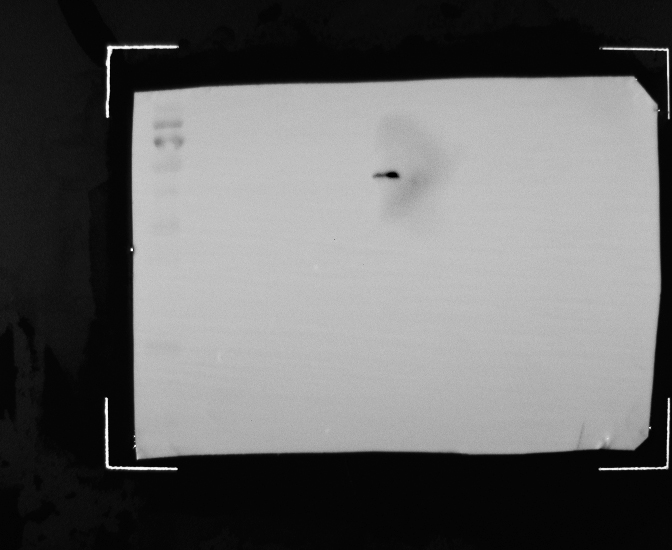


Figure S4. The raw image of binding activity of the recombinant VLR-L and VLR2-S3 to *Vibrio alginolyticus* and *Corynebacterium glutamicum*.


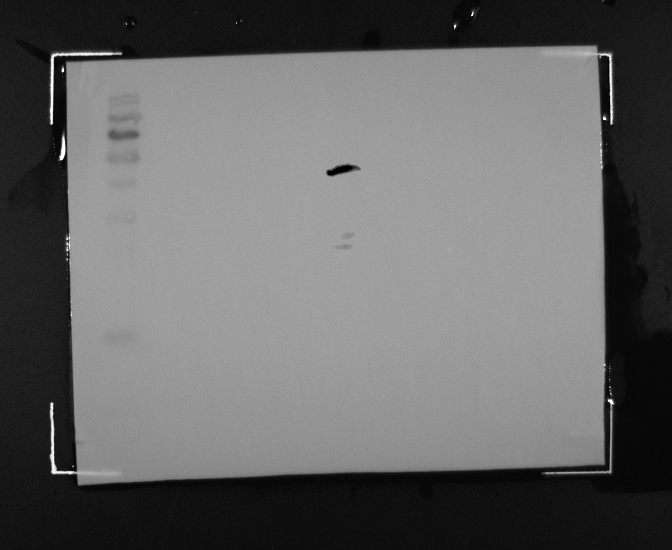


Figure S5. The raw image of binding activity of the recombinant VLR-L and VLR2-S3 to *Vibrio harveyi* and *Micrococcus lysodeikticus*.


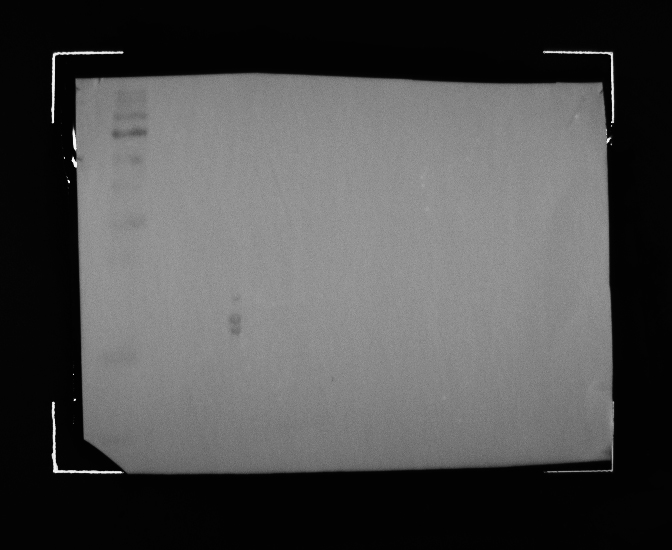


Figure S6. The raw image of binding activity of the recombinant VLR2-S2 to *Vibrio parahaemolyticus*, *Staphylococcus aureus*, *Vibrio alginolyticus* and *Corynebacterium glutamicum*.


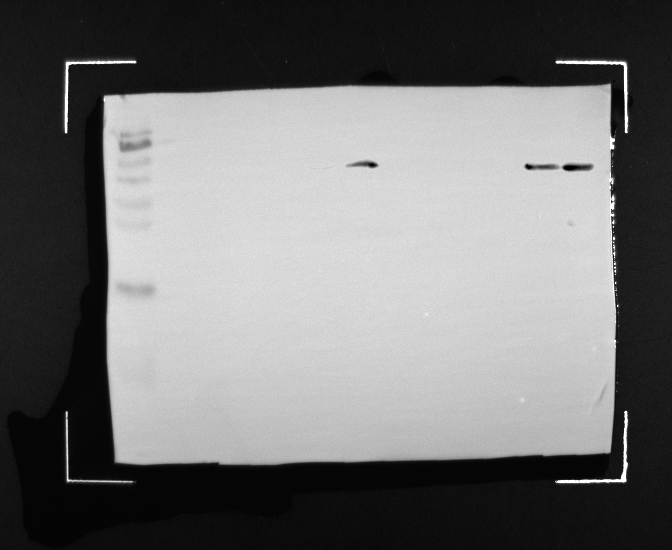


Figure S7. The raw image of binding activity of the recombinant VLR2-S2 to *Vibrio harveyi* and *Micrococcus lysodeikticus*; VLR2-S5 to *Vibrio parahaemolyticus* and *Staphylococcus aureus*.


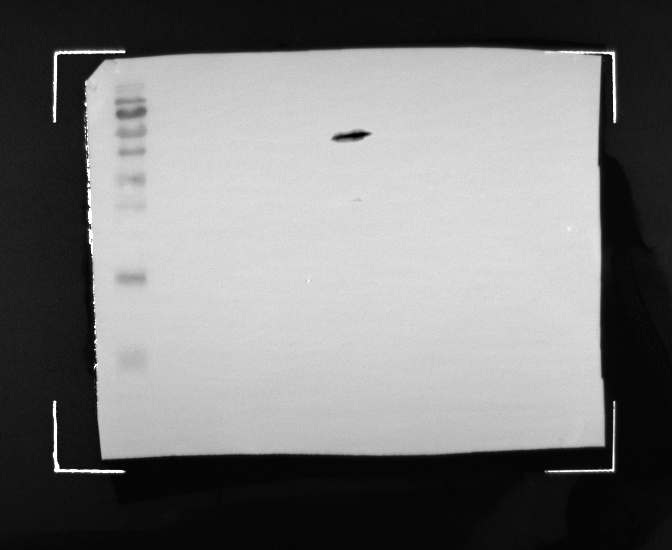


Figure S8. The raw image of binding activity of the recombinant VLR2-S5 to *Vibrio alginolyticus*, *Corynebacterium glutamicu*, *Vibrio harveyi* and *Micrococcus lysodeikticus*.


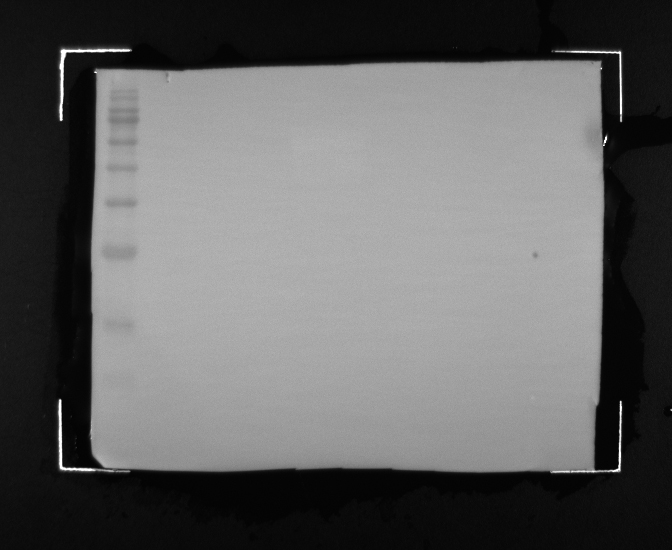


Figure S9. The raw image of binding activity of the recombinant VLR2-S8 to *Vibrio parahaemolyticus*, *Staphylococcus aureus*, *Vibrio alginolyticus* and *Corynebacterium glutamicum*.


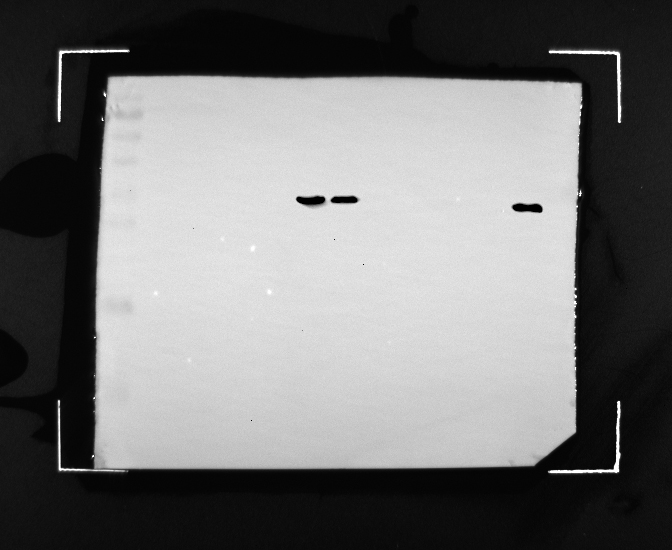


Figure S10. The raw image of binding activity of the recombinant VLR2-S8 to *Vibrio harveyi* and *Micrococcus lysodeikticus*.


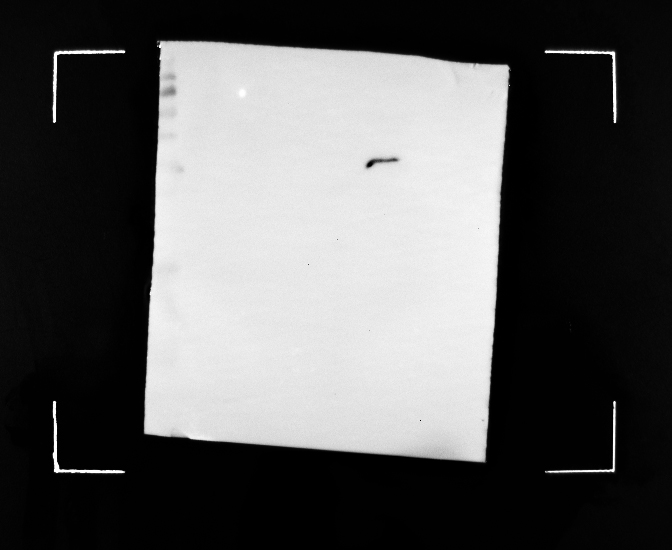


Figure S11. The raw image of binding activity of the recombinant VLR-S9 and pET-32a to *Vibrio parahaemolyticus* and *Staphylococcus aureus*.


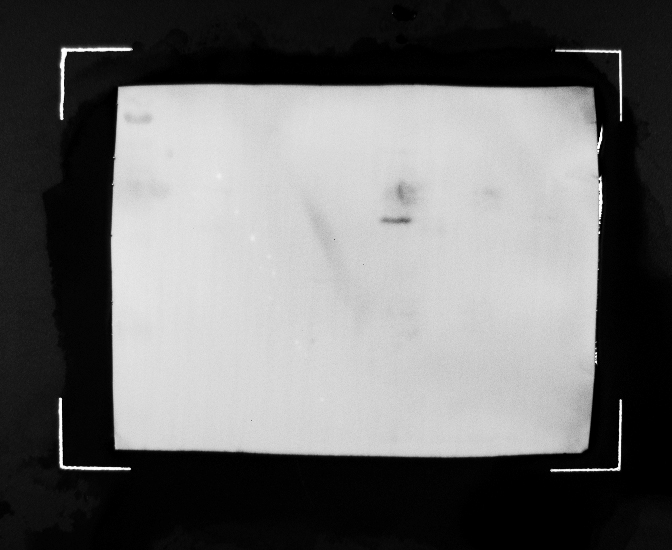


Figure S12. The raw image of binding activity of the recombinant VLR-S9 and pET-32a to *Vibrio alginolyticus* and *Corynebacterium glutamicum*.


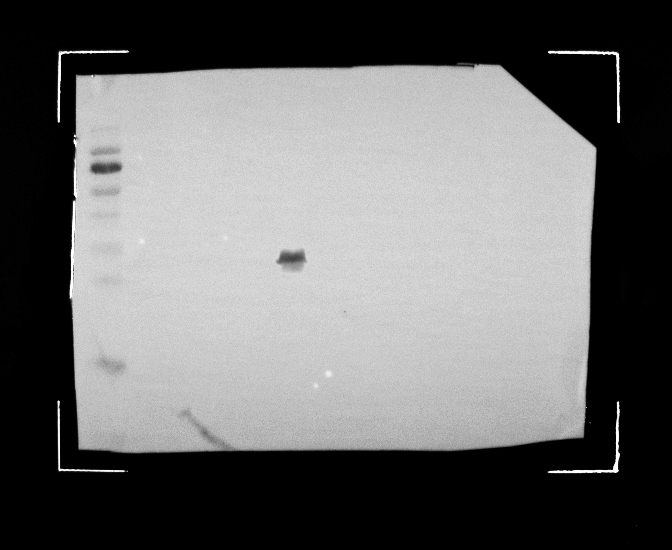


Figure S13. The raw image of binding activity of the recombinant VLR-S9 and pET-32a to *Vibrio harveyi* and *Micrococcus lysodeikticus*.


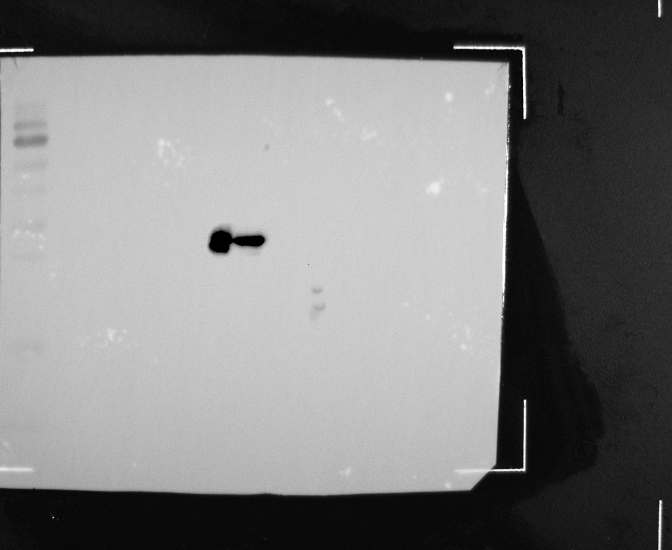

Supplement: Supplementary file 1 [file DataSheet_1.docx]
